# Antibiotic-Potentiating Effect of Some Bioactive Natural Products against Planktonic Cells, Biofilms, and Virulence Factors of *Pseudomonas aeruginosa*

**DOI:** 10.1155/2023/9410609

**Published:** 2023-08-23

**Authors:** Larissa Yetendje Chimi, Borel Ndezo Bisso, Guy Sedar Singor Njateng, Jean Paul Dzoyem

**Affiliations:** Department of Biochemistry, Faculty of Science, University of Dschang, Dschang, Cameroon

## Abstract

**Background:**

*Pseudomonas aeruginosa* is an opportunistic human pathogen that causes infections that are mediated by both virulence factor production and biofilm formation. In addition, many antibiotics are increasingly losing their efficacy due to the development of resistance. The screening of potentially bioactive natural compounds that have both antivirulence and antibiofilm activities to enhance antibiotic efficacy and reverse antibiotic resistance is a good strategy to overcome these issues. In this study, the antibacterial, antibiofilm, and antivirulence factor activities of some bioactive natural products in combination with conventional antibiotics were evaluated against clinical isolates of *P. aeruginosa*.

**Methods:**

The broth microdilution method was used to determine the antibacterial and antibiofilm activities. The checkerboard method was used to evaluate the combination interactions. Spectrophotometric and agar plate techniques were used to assess the effect of the combination on the pyocyanin production and the motility in *P. aeruginosa* ATCC 27853 strain.

**Results:**

Out of the eighteen combinations tested, ten exhibited synergistic effects against planktonic cells, seven against biofilm inhibition, and five against the eradication of mature biofilm of *P. aeruginosa* biofilm. The best synergistic effect was the association of amikacin and sinapic acid against planktonic cells (FICI = 0.08) with a 70-fold reduction in the MIC value of amikacin. The same combination showed significant synergistic inhibition of biofilm formation (FICI = 0.1) and biofilm eradication (FICI = 0.15) reducing the MBIC and MBEC of amikacin by 32-fold. Some selected synergistic combinations showed statistically significant differences (*p* < 0.01 or *p* < 0.001) in the inhibition of virulence factors compared to the antimicrobials alone.

**Conclusion:**

In summary, this study revealed sinapic acid as an antibiotic adjuvant and antivirulence compound to overcome *P. aeruginosa* infections. This finding indicates that the combinations of amikacin plus sinapic acid, ceftazidime plus thymol, and norfloxacin plus curcumin could be considered promising candidates for the development of combination therapies targeting virulence factors against *P. aeruginosa* infections.

## 1. Introduction

Bacterial infections caused by invading pathogenic bacteria or opportunistic pathogens such as *P. aeruginosa* are major infectious diseases worldwide causing many diseases [1]. These infections include cystic fibrosis, pneumonia, otitis, endophthalmitis, endocarditis, urinary tract infections, and soft tissue infections [2]. These infections caused by *P. aeruginosa* are associated with high morbidity and mortality rates since *P. aeruginosa* is capable of surviving in a wide range of environments [3]. Aminoglycosides, *β*-lactams, and fluoroquinolones are classes of conventional antibiotics commonly used in the treatment of *P. aeruginosa* infections. However, resistance to these drugs has led to the emergence of new *P. aeruginosa* infectious diseases [4]. Despite the difference in both processes, virulence factors are necessary for the development of antibiotic resistance by enabling pathogenic bacteria to overcome antimicrobial therapies and to survive and adapt to competitive environments [5]. *P. aeruginosa* can adapt to the hosts by secreting a variety of virulence factors such as pyocyanin production, swarming and swimming motilities, and biofilm formation which contributes to successful infection causing disease and confer bacterial communication and drug resistance [6]. Pyocyanin has been well documented as an important virulence factor given the antimicrobial resistance and chronic nature of pseudomonal infections [7]. Swimming involves the rotation of a single polar flagellum. Swarming motility requires multicellular coordination of bacteria across mucosal sites and has been linked to increased antibiotic resistance while twitching motility involves bacterial attachment and initial colonization of mucosal cell surfaces [8]. Biofilms are highly structured surface-associated microbial cells that establish a connection between themselves and are embedded in an extracellular polymeric matrix [9, 10]. Biofilm represents an important virulence factor in helping *P. aeruginosa* to escape the host immune defense mechanisms and has the ability to protect the bacteria from antibiotics [11]. Hence, the MIC of an antimicrobial that is effective on sessile bacteria is 10 to 1000 times more concentrated than that of the one which would be active on their planktonic cells [12]. To overcome multidrug resistance and biofilm issues of *P. aeruginosa*, it is necessary to find new therapeutic strategies.

Natural products have been an important source of new pharmacological compounds, and interest in natural products as drug leads is currently being revitalized, particularly for tackling antimicrobial resistance [13, 14]. The natural product-based drug combination is now attracting scientists worldwide since they have the advantage of treating complex diseases by regulating multiple targets, enhancing the sensitivity of conventional therapy, reversing drug resistance, and lowering the effective dose of their associated therapy [15]. Synergistic effects of natural products in combination with antibiotics against microbial pathogens have been previously reported [16–18].

A diverse set of natural products including flavonoids (curcumin, quercetin), quinones (plumbagin), alkaloids (piperine), triterpenoids, and essential oil phenols (eugenol and thymol) has been reported to be pharmacologically active [13]. Some of them have been proven to be effective as antibacterial and antibiofilm agents, in suppressing cell adhesion and attachment, decreasing the virulence factors' production, inhibiting the formation of the polymer matrix, and thereby blocking the quorum sensing network [19]. Therefore, we hypothesized that their combination with antibiotics that are losing efficacy due to the spread of resistance to target virulence factors could be more promising to overcome these issues. The main objective of this study is to investigate the effect of the combination of some bioactive natural products (curcumin, piperine, plumbagin, thymol, quercetin, and sinapic acid) with antibiotics (amikacin, ceftazidime, and norfloxacin) against planktonic cells and virulence factors of *P. aeruginosa* isolates.

## 2. Materials and Methods

### 2.1. Chemicals and Natural Products

Dimethyl sulfoxide (DMSO), p-iodonitrotetrazolium chloride (INT), and 3 (4, 5-dimethylthiazole-2-yl)-2, 5-diphenyltetrazolium bromide (MTT) were also purchased from Sigma-Aldrich. The Mueller-Hinton agar (MHA) and Mueller-Hinton broth (MHB) were purchased from Dominique Dutscher SAS, France. The Luria Bertani (LB) broth was purchased from Merck, Germany. Six antibiotics belonging to five classes selected based on their activity against *P. aeruginosa* were used. These included four aminoglycosides (amikacin, neomycin, paromomycin, and streptomycin), one cephalosporin (ceftazidime), and one quinolone (norfloxacin). Six natural products selected based on the diversity of their biological activity were used. They included curcumin, piperine, plumbagin, thymol, quercetin, and sinapic acid. All antibiotics and natural products were purchased from Merck, Germany.

### 2.2. Antibacterial and Antibiofilm Activity

#### 2.2.1. Bacterial Isolates and Culture Conditions

Thirty-seven clinical isolates of *P. aeruginosa* collected from infected wounds were used. In addition, one reference strain obtained from the American Type Culture Collection (ATCC 27853) was used as a control. They were maintained on the Mueller-Hinton agar slant at 4°C and subcultured on a fresh appropriate agar plate 24 hours before the antibiofilm assay. All thirty-eight *P. aeruginosa* have been previously assessed for their ability to express the investigated virulence factors (unpublished data available upon request).

#### 2.2.2. Minimum Inhibitory Concentration (MIC) and Minimum Bactericidal Concentration (MBC) Determination

The antimicrobial susceptibility of antibiotics and the antibacterial activity of natural products against the planktonic cells of the 37 isolates of *P. aeruginosa* were carried out by determining the MIC and MBC parameters. This was performed according to the broth microdilution method described by Dzoyem et al. [20]. Results were expressed as MIC and MBC ranges and geometric means of the 38 isolates screened.

#### 2.2.3. Minimum Biofilm Inhibitory Concentration (MBIC) and Minimum Biofilm Eradication Concentration (MBEC) Determination

The MBIC and MBEC values of antibiotics and natural products were evaluated against the selected best biofilm former isolates (19 including the reference strain) of *P. aeruginosa*. This was performed by the microtiter plate method as described by Ndezo Bisso et al. and modified by Klrmusaoaylu and Kaşlkçl [21, 22]. For MBIC determination, 100 *μ*L of serial twofold dilutions of natural product or antibiotic (at concentrations ranging from 32 to 4096 *μ*g/mL) and 100 *μ*L of bacterial inoculum (1.5 × 10^6^ CFU/mL) were introduced in the microplate followed by incubation at 37°C for 24 h. After incubation, the microtiter plate was removed and washed three times with phosphate-buffered saline (PBS). Then, 200 *μ*L of MTT solution (0.5 mg/mL) was added to each well followed by incubation at 37°C for 4 h. After incubation, MTT was removed and 200 *μ*L of DMSO was added to the wells to dissolve the formed formazan crystals. Wells without potential antibacterial agents were used as positive controls while wells containing only broth were used as blanks. Metabolic activity was quantified by measuring the fluorescence at 570 nm using a microtiter plate reader. The lowest concentration of natural product or antibiotic causing 100% inhibition of metabolic activity was recorded as MBIC.

For MBEC determination, the preformed biofilms were treated with 200 *μ*L of serial twofold dilutions of natural product or antibiotic (at concentrations ranging from 32 to 4096 *μ*g/mL) followed by incubation at 37°C for 24 h. After incubation, the plates were treated as described above, and the MBEC was defined as the lowest concentration of natural product or antibiotic reducing 100% of metabolic activity. Results were expressed as MBIC and MBEC ranges and geometric means of the 19 isolates screened.

### 2.3. Checkerboard Assay for Combination Studies

Referring to the MIC values of the selected antibiotics and natural compounds, the checkerboard assay was designed to determine their FICIs in combination against the nineteen best biofilm formers of *P. aeruginosa* isolates. The three antibiotics having the lowest MIC mean values were used (amikacin, ceftazidime, and norfloxacin).

#### 2.3.1. Assessment of Interaction between Antibiotics and Natural Products against Planktonic Cells

The effect of the combination of antibiotics with natural substances on planktonic cells was assessed by the checkerboard method as previously reported [21]. Briefly, in two microtiter plates, 50 *μ*L of MHB was introduced. In the first plate, 50 *μ*L of the antibiotic solution was added to all the wells of the first column followed by a serial dilution. In the second plate, 50 *μ*L of natural substance solution was added to all the wells of the first row, and dilutions were made as described for the first plate. At the end of the dilution, the content of one of the plates was added to the second, respectively, to the position of the wells. Thereafter, 100 *μ*L of bacterial inoculum (1.5 × 10^6^ CFU/mL) was introduced into the wells except for the neutral control wells. The plates were incubated at 37°C for 24 hours. The INT was used as an indicator of the bacterial growth, and the fractional inhibitory concentration index (FICI) was calculated to evaluate the combination interaction as follows: FICI = (MIC of antibiotic in the combination/MIC of antibiotic alone) + (MIC of natural product in the combination/MIC of natural products alone). The interaction was classified as follows: synergy when FICI ≤ 0.5, additivity when 0.5 < FICI ≤ 1, indifference when 1 < FICI ≤ 4, and antagonism when FICI > 4 [23].

#### 2.3.2. Assessment of Interaction between Antibiotics and Natural Products against the Biofilm Formation

The effect of the combination of antibiotics and natural products to prevent biofilm formation was evaluated by the checkerboard method as previously described [21]. The experiment was carried out with MHB supplemented with 1% glucose. After incubation, the plates were emptied of their contents and washed three times with phosphate-buffered saline (PBS), and the metabolic activity of biofilm was quantified using the MTT assay as described above. The lowest concentration of antibiotics or natural products that reduces the metabolic activity of biofilm by 100% was considered as the minimal biofilm inhibitory concentration (MBIC). The well containing MHB without bacteria was used as blank while the wells containing bacteria and MHB supplemented with 1% glucose were used as the positive controls. The fractional inhibitory concentration index (FICI) was determined as described above.

#### 2.3.3. Assessment of Interaction between Antibiotics and Natural Products against Mature Biofilm

The effect of the combination of antibiotics with natural products to eradicate the mature biofilm was carried out according to the checkerboard method described above except that the biofilm was formed before the antimicrobial treatment. The metabolic activity biofilm was quantified with MTT assay as described above. Then, the minimal biofilm eradicating concentration (MBEC) of the antibiotics and substances was determined as described above. The effect of the association was determined after the calculation and interpretation of FICI values as described above.

### 2.4. Effect of Synergistic Combinations on Virulence Factor Expression

#### 2.4.1. Effect of Synergistic Combination on Pyocyanin Production

The inhibition of the production of pyocyanin by the most potent synergistic combinations was carried out on the strain of *P. aeruginosa* ATCC 27853 according to the method described by Alayande et al. with slight modifications [24]. Briefly, 10 mL of bacterial suspension (1 × 10^8^ CFU/mL) in LB medium was treated with antibiotics or natural substances, alone and in combination, and then incubated at 37°C for 72 hours. After incubation, the cultured cells were centrifuged at 1500 × *g* for 10 min, then 5 mL of the supernatant was added to 3 mL of chloroform, and the mixture was agitated vigorously using a vortex mixer. The chloroform layer was collected and added to 1 mL of 0.2 M of HCl and then centrifuged at 1500 × *g* for 10 min. The optical density of the HCl layer was measured at 520 nm using a spectrophotometer (Biobase BK-D590 Double Beam Scanning UV/Vis, China). The solution of HCl (0.2 M) was used as blank control. The concentration of pyocyanin was obtained using the following formula: concentration of pyocyanin (*μ*g/mL) = (OD_520nm_ − OD_blank_) × 17.072.

#### 2.4.2. Effect of Synergistic Combination on the Inhibition of *P. aeruginosa* Motility

The inhibition of motility in *P. aeruginosa* ATCC 27853 by selected synergistic combinations (combination with the lowest FICI either on planktonic cell or biofilm inhibition) was carried out using the method described by Abu El-Wafa et al., with some modifications [25]. Briefly, the tested samples were prepared at their minimum inhibitory concentration obtained after combination and added, alone or in combination, to LB broth containing 1% (for swarming) or 0.5% (for swimming) agar before being poured into the Petri dishes. After drying, 2.5 *μ*L of bacterial inoculum was gently placed in the center of the Petri dishes (swarming) or was stabbed into the agar medium (swimming). Then, the plates were incubated at 37°C for 72 hours. After incubation, the diameter of the migration area produced by the bacterial strain was measured (mm).

### 2.5. Statistical Analysis

The geometric means of MICs, MBCs, MBICs, and MBECs were calculated using Microsoft Excel 2016. For the effect of synergistic combinations on virulence factors, differences between means of single treatment and combination were assessed by two-way ANOVA followed by Dunnett's test using GraphPad Prism 8.

## 3. Results

### 3.1. Antibacterial and Antibiofilm Activity of Natural Products and Antibiotics

The MIC and MBC ranges and geometric means of antibiotics and natural products against 37 isolates of *P. aeruginosa* as well as the MBIC and MBEC ranges and geometric means against 19 biofilm former isolates of *P. aeruginosa* are presented in Table 1. The results revealed that of all antibiotics tested, amikacin showed the best activity against planktonic cells and biofilm of *P. aeruginosa* with an average MIC of 7.08 *μ*g/mL and MBC of 45.68 *μ*g/mL, while the average MBIC and MBEC obtained were 48.47 *μ*g/mL and 330.11 *μ*g/mL, respectively. Among the natural products, sinapic acid showed the most potent antibacterial activity with an average MIC of 27.79 *μ*g/mL and an average MBC of 370.53 *μ*g/mL. Quercetin had an antibiofilm activity with an average MBIC equal to 52.21 *μ*g/mL and an MBEC equal to 195.37 *μ*g/mL.

### 3.2. Effect of the Combination of Antibiotics with Natural Products against Planktonic Cells

Table 2 shows the results of the combinations between the antibiotics which have shown the best inhibitory activity and the natural substances against the planktonic cells of *P. aeruginosa*.

The results are presented as the MIC and FIC means of 19 isolates of *P. aeruginosa*. It emerges from Table 2 that three types of interactions were obtained: synergy, additivity, and indifference. No antagonistic interaction was observed. Out of the eighteen combinations tested, ten synergies were recorded with FICI values between 0.08 and 0.47. Six additivities were observed with FICIs between 0.65 and 0.99, while two indifferences were observed with FICIs between 1.04 and 1.05. The best synergistic effect was observed in the association of amikacin and sinapic acid with a FICI value of 0.08 and a 70-fold reduction of the MIC of amikacin. This was immediately followed by the synergistic association of norfloxacin and curcumin with a FICI value of 0.17 and a 30.74-fold reduction of the MIC of norfloxacin.

### 3.3. Effect of the Combination of Antibiotics with Natural Products to Prevent Biofilm Formation

The result of the interaction obtained from the combinations of antibiotics and natural products against the biofilm formation is shown in Table 3. The results are presented as MBIC and FIC means of 19 isolates of *P. aeruginosa*. From this table, it emerges that out of eighteen combinations tested, seven synergies were obtained with FICI values ranging between 0.10 and 0.41. Nine combinations showed additivities with FICIs ranging between 0.51 and 0.88 while two combinations presented indifferences with FICIs between 1.14 and 1.16. No antagonism was observed. The association of amikacin and sinapic acid appears as the best synergistic combination with a FICI value of 0.1 and a 32-fold reduction in MBIC of amikacin. Another good synergistic combination was also obtained with norfloxacin, curcumin, and sinapic acid with FICI values of 0.15 and 0.27, respectively.

### 3.4. Effect of the Combination of Antibiotics and Natural Product to Eradicate Mature Biofilm

Table 4 shows the results obtained from the combination of antibiotics with natural products against the eradication of preformed or mature biofilm. A perusal of this table shows that out of eighteen combinations tested, only five synergy interactions were obtained, with FICI values varying between 0.15 and 0.47. The best synergistic interaction was shown by the combination of amikacin and sinapic acid, with a FICI value of 0.15 and a 32-fold reduction of the MBEC value of amikacin. This was followed by the synergic effect of the norfloxacin and curcumin combination which had a FICI value of 0.18 with an 18-fold reduction of the MBEC value of norfloxacin.

### 3.5. Effect of Synergistic Combinations on the Inhibition of Pyocyanin Production and Swarming and Swimming Motilities

**Figure 1:**
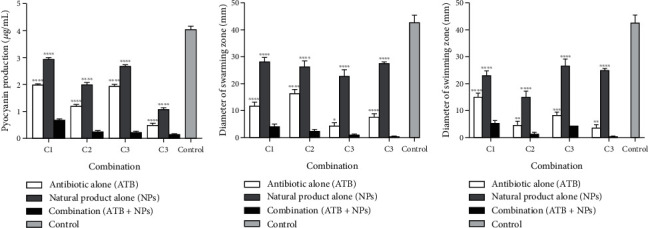
Effect of selected combination on (a) pyocyanin production, (b) swarming motility, and (c) swimming motility in *Pseudomonas aeruginosa* strain ATCC 27853. C1: ceftazidime (1.95 *μ*g/mL)+thymol (165.05 *μ*g/mL); C2: amikacin (0.16 *μ*g/mL)+sinapic acid (2.20 *μ*g/mL); C3: norfloxacin (0.38 *μ*g/mL)+curcumin (88.42 *μ*g/mL); C4: amikacin (1.38 *μ*g/mL)+sinapic acid (5.37 *μ*g/mL). Statistical analysis was performed with Dunnett's multiple comparison test using two-way ANOVA; ^∗^*p* < 0.05 and ^∗∗∗∗^*p* < 0.0001 between the single treatment and the combination.

Following the combination tests, the best synergistic combinations against planktonic cells (FICI ≤ 0.2), biofilm inhibition (FICI ≤ 0.3), and biofilm eradication (FICI ≤ 0.4) were selected for the assessment of their effect on virulence factors. In addition, the combinations selected were those for which the MIC value of the antimicrobial in the combination was lower than the value of its MIC alone against *P. aeruginosa* ATCC 27853. These conditions allowed us to select four combinations, namely, C1: ceftazidime (1.95 *μ*g/mL)+thymol (165.05 *μ*g/mL), C2: amikacin (0.16 *μ*g/mL)+sinapic acid (2.20 *μ*g/mL), C3: norfloxacin (0.38 *μ*g/mL)+curcumin (88.42 *μ*g/mL), and C4: amikacin (1.38 *μ*g/mL)+sinapic acid (5.37 *μ*g/mL). The results of the potential of the selected combinations to inhibit the production of pyocyanin, as well as the swarming and swimming motilities in *P. aeruginosa* ATCC 27853, are shown in Figures 1(a)–1(c). The effect of the antimicrobial combination was compared to that of each antimicrobial alone.  Figure 1 shows a synergistic effect of the selected combination against the investigated virulence factors.  Figure 1(a) shows that in the C3 combination, the pyocyanin value dropped from 1.95 *μ*g/mL and 2.67 *μ*g/mL, respectively, with ceftazidime and thymol alone to 0.22 *μ*g/mL in the combination. The C3 and CBI combinations inhibited the diameter of swimming motility below 0.5 mm.

Figures 1(b) and 1(c) show that ceftazidime, amikacin, and norfloxacin alone significantly inhibited the motility of *P. aeruginosa* ATCC 27853 with the inhibition of diameters between 3.66 mm and 15.00 mm for swarming and between 7.66 mm and 11.66 mm for swimming. Compared to antibiotics, thymol, curcumin, and sinapic acid alone weakly inhibited both motilities with the diameters of the swarming motility zones being between 27.33 and 28.00 mm, than that of swimming between 23.00 mm and 25.00 mm. All the combinations showed a synergistic inhibitory effect on both swarming and swimming motilities. Overall selected synergistic combinations showed statistically significant differences (*p* < 0.01 or *p* < 0.001) for the inhibition of all virulence factors compared to the antimicrobials alone.

## 4. Discussion

*P. aeruginosa* has become a major threat to human health due to the constant emergence of drug-resistant strains and the diversity of virulence factors exploited in its pathogenic process. Besides, many antibiotics are increasingly losing their efficacy due to the development of resistance. Therefore, the screening of potentially bioactive natural compounds that have both antivirulence and antibiofilm activities to enhance antibiotic efficacy and reverse resistance is an interesting alternative to alleviate these issues. In this study, the antibacterial and antibiofilm activities of some bioactive natural products alone and in combination with conventional antibiotics were evaluated against clinical isolates of *P. aeruginosa*. Then, the antivirulence factor effect of selected synergistic combinations was assessed in *P. aeruginosa* ATCC 27853.

The antibacterial and antibiofilm activities of six antibiotics (amikacin, paromomycin, streptomycin, neomycin, ceftazidime, and norfloxacin) and six bioactive natural products (curcumin, piperine, plumbagin, thymol, quercetin, and sinapic acid) against 38 isolates of *P. aeruginosa* were determined. It is noteworthy that we previously reported the antibiotic resistance profile of the *P. aeruginosa* isolates used in this study (unpublished data available upon request). Therefore, the MIC values recorded were mainly intended to be used in the checkerboard method for combination studies. All the investigated bioactive natural products showed antibacterial activity. Sinapic acid was the most potent followed by curcumin and quercetin. In fact, these natural products have bactericidal effects on bacterial pathogens through several mechanisms such as destabilization of the bacterial membrane, inhibition of enzymes produced by bacteria, nucleic acid synthesis inhibition, and suppression of bacterial biofilm formation [26]. This result is in agreement with the literature since they were selected based on the wide range of their pharmacological properties including antimicrobial, antioxidant, anti-inflammatory, and anticancer activities [27]. Similarly, all the investigated antibiotics and natural products showed antibiofilm activity. It was observed that concentrations needed to eradicate the mature biofilm were usually higher than those needed to inhibit the biofilm formation or to inhibit the growth of planktonic cells. This finding follows the general trend according to which the antimicrobial concentration killing microorganisms in a biofilm including the persister cells, low penetration of antimicrobial agents into the biofilm through the extracellular matrix, low metabolic state at the base of the biofilm, high transmission of resistance genes, and overexpression of efflux pumps in the biofilm cells can be hundreds to thousands of times higher than the MIC for the same antimicrobial to be effective [18, 28].

The three antibiotics that showed the best inhibitory activity against the planktonic cells were selected for combination with the six natural products used. Thus, eighteen combinations were evaluated against planktonic cells, biofilm formation, and biofilm eradication of *P. aeruginosa*. Out of the eighteen combinations tested against each of the three forms of *P. aeruginosa*, ten exhibited synergistic effects against planktonic cells, seven showed synergistic effects against the biofilm inhibition, and five had a synergistic effect against the eradication of mature biofilm.

These synergistic effects found on planktonic cells could be attributed to the ability of the studied natural products (sinapic acid, thymol, and curcumin) to disrupt the bacterial membrane that causes rapid depolarization, facilitating the influx of antibiotics inside bacterial cell. In contrast, the synergistic effects found on *P. aeruginosa* biofilms may be due to pyocyanin inhibition by sinapic acid, thymol, and curcumin, resulting in inhibition of biofilm formation and thus increasing the antibiotic efficacy [29]. This result highlights the recalcitrant nature of biofilms to antimicrobials. The recalcitrance of biofilms to antimicrobials is well documented as part of the definition of biofilms, and it is increasing with biofilm maturation [30]. The antibiotic tolerance of biofilms has been attributed to the expression of biofilm-specific genes, restricted penetration of the antibiotics, the presence of persisters, and restricted growth at low oxygen tension [31].

Three synergistic combinations significantly enhanced the activity of the antibiotic against planktonic cells. These were the combination of ceftazidime with thymol (31-fold reduction of the antibiotic MIC), amikacin with sinapic acid (70-fold reduction of the antibiotic MIC), and norfloxacin with curcumin (30-fold reduction of the antibiotic MIC). Only the combination of amikacin with sinapic acid significantly enhanced the activity of the antibiotic against both the biofilm formation and the biofilm eradication with 32- and 31-fold reduction of the amikacin MIC value. Several mechanisms have been proposed to explain the synergistic action of phytochemicals with antibiotics. Several compounds have been reported to act synergistically with existing antibiotics to improve the activity of these drugs, and significant decreases in the MICs of several antibiotics in combination with natural products have been observed [16]. It appears from this work that only the combination of amikacin with sinapic acid was synergistically effective against planktonic cells, biofilm formation, and biofilm eradication of *P. aeruginosa*. In addition, this combination was the most potent against planktonic cells. Amikacin like other aminoglycoside antibiotics binds to the bacterial ribosomal subunits inhibiting the protein synthesis, while sinapic acid is a naturally occurring plant phenolic compound with potential antibacterial, antioxidant, anticancer, and anti-inflammatory activities [32]. To the best of our knowledge, this is the first study reporting the synergistic effect of sinapic acid with antibiotics against *P. aeruginosa*.

However, a synergistic interaction between *Staphylococcus aureus* and *Escherichia coli* has been observed between antibiotics and extracts from grape pomace that contains a high concentration of phenolic compounds including sinapic acid [33]. Although no study reporting the pharmacological mechanism of action of sinapic acid was found in the literature, its synergistic action could be similar to that of other bioactive phytochemicals. Natural compounds can exert their synergistic action through several strategies, such as the inhibition of target modifying and drug-degrading enzymes such as efflux pumps or by facilitating their entry into the cell by altering the cytoplasmic membrane or dispersing biofilms [34]. Some of the synergistic interactions between natural products and antibiotics include reduced antibiotic doses and side effects, or increased efficiency, stability, and bioavailability [35]. The antivirulence effect of the selected best synergistic combination evaluated against the production of pyocyanin and the motility (swarming and swimming) in *P. aeruginosa* ATCC 27853 revealed significant inhibition compared to the substances taken individually.

The combination of amikacin with sinapic acid was the most active against the three virulence factors evaluated. Although our literature search did show any previous report on the antivirulence activity of sinapic acid, the antivirulence effects of some drug candidates including natural compounds on *P. aeruginosa* have been previously evaluated [36].

## 5. Conclusion

This work showed a prominent synergistic effect of ceftazidime, amikacin, and norfloxacin in combination, respectively, with thymol, sinapic acid, and curcumin against planktonic cells of *P. aeruginosa* and between amikacin and sinapic acid against the inhibition of biofilm formation of *P. aeruginosa*. These combinations also significantly reduced the expression of virulence factors including the production of pyocyanin and motility. The best synergistic effect was made up of amikacin and sinapic acid with up to a 70-fold reduction in the MIC of amikacin against *P. aeruginosa*. This finding indicates that the combinations of amikacin plus sinapic acid, ceftazidime plus thymol, and norfloxacin plus curcumin could be considered good candidates for promising combination therapies targeting virulence factors against *P. aeruginosa* infections.

## Figures and Tables

**Table 1:** Minimum inhibitory concentration (MIC), minimum bactericidal concentration (MBC), minimum biofilm inhibitory concentration (MBIC), and minimum biofilm eradication concentration (MBEC) ranges and geometric means of antibiotics and natural products against 37 or 19 isolates of *P. aeruginosa*.

Antimicrobial agents	MIC^†^		MBC^†^		MBIC^††^		MBEC^††^	
	Range	G-mean	Range	G-mean	Range	G-mean	Range	G-mean
Amikacin	1-16	7.08	8-128	45.68	16-128	45.47	64-512	330.11
Paromomycin	>256	-	>256	-	256-1024	592.84	>2048	-
Streptomycin	>256	-	>256	-	256-1024	565.89	>2048	-
Neomycin	8-128	67.69	>256	-	256-1024	592.84	>2048	-
Ceftazidime	2-128	28	16-128	56.73	64-256	175.16	512-1024	815.16
Norfloxacin	2-16	7.47	16-128	48.43	16-256	106.95	256-1024	619.79
Curcumin	64-1024	417.68	512-1024	905.85	128-2048	565.89	>2048	-
Piperine	>1024	-	>1024	-	>2048	-	>2048	-
Plumbagin	128-1024	644.92	>1024	-	1024-2048	-	>2048	-
Thymol	>1024	-	>1024	-	256-1024	464.84	>2048	-
Quercetin	32-128	77.47	128-1024	494.93	16-128	52.21	128-256	195.37
Sinapic acid	16-64	27.79	128-1024	370.53	32-128	74.11	128-512	303.16

^†^Against 38 isolates. ^††^Against 19 isolates. G-mean: geometric mean; -: not determined.

**Table 2:** Mean of the minimum inhibitory concentration (MIC), fractional inhibitory concentration (FIC), and fractional inhibitory concentration index (FICI) of antibiotics (ATB) and natural products (NPs) in combination against nineteen *P. aeruginosa* isolates.

Antimicrobial agent combination	MIC^†^ (*μ*g/mL)				FIC		MIC reduction fold of ATB	FICI/interpretation
	Alone		Combined					
	ATB	NPs	ATB	NPs	ATB	NPs		
Cef+Cur	54.12	640	11.20	280.42	0.21	0.44	4.83	0.65/A
Cef+Pip	61.89	>1024	21.68	485.05	0.35	0.47	2.85	0.82/A
Cef+Plu	61.89	>1024	29.68	500	0.48	0.49	2.09	0.97/A
Cef+Thy	61.89	>1024	1.95	165.05	0.03	0.16	31.74	0.19/S
Cef+Quer	61.89	97.68	12.95	23.58	0.21	0.24	4.78	0.45/S
Cef+Sin A	61.89	35.37	9.37	5.42	0.15	0.15	6.61	0.30/S
Ami+Cur	11.26	640	2.20	176.84	0.20	0.28	5.12	0.47/S
Ami+Pip	11.26	>1024	5.68	498.53	0.50	0.49	1.98	0.99/A
Ami+Plu	11.26	>1024	6.50	471.58	0.58	0.46	1.73	1.04/I
Ami+Thy	11.26	>1024	2.90	217.26	0.26	0.21	3.88	0.47/S
Ami+Que	11.26	97.68	2.03	17.89	0.18	0.18	5.55	0.36/S
Ami+Sin A	11.26	35.37	0.16	2.20	0.01	0.06	70.38	0.08/S
Nor+Cur	11.68	660.21	0.38	88.42	0.03	0.13	30.74	0.17/S
Nor+Pip	11.68	>1024	6.37	519.58	0.55	0.51	1.83	1.05/I
Nor+Plu	11.68	>1024	7.68	326.74	0.66	0.32	1.52	0.98/A
Nor+Thy	11.68	>1024	3.25	377.26	0.28	0.37	3.59	0.65/A
Nor+Que	11.68	97.68	1.55	26.95	0.13	0.28	7.54	0.41/S
Nor+Sin A	11.68	35.37	0.58	6.05	0.05	0.17	20.14	0.22/S

^†^Geometric mean value of 19 isolates. Cef: ceftazidime; Ami: amikacin; Nor: norfloxacin; Cur: curcumin; Pip: piperine; Plu: plumbagin; Thy: thymol; Que: quercetin; Sin A: sinapic acid; S: synergy; A: additivity; I: indifference.

**Table 3:** Mean of the minimum biofilm inhibitory concentration (MBIC), fractional inhibitory concentration (FIC), and fractional inhibitory concentration index (FICI) of antibiotics (ATB) and natural products (NPs) in combination against nineteen *P. aeruginosa* isolates.

Antimicrobial agent combination	MBIC (*μ*g/mL)				FIC		MBIC reduction fold of ATB	FICI/interpretation
	Alone		Combined					
	ATB	NPs	ATB	NPs	ATB	NPs		
Cef+Cur	175.16	566.21	43.79	168.42	0.25	0.30	4.00	0.55/A
Cef+Pip	175.16	>2048	78.32	592.84	0.45	0.29	2.24	0.74/A
Cef+Plu	175.16	>1280	64.84	646.74	0.37	0.51	2.70	0.88/A
Cef+Thy	175.16	464.84	50.95	148.21	0.29	0.32	3.44	0.61/A
Cef+Que	175.16	52.21	73.68	22.32	0.42	0.43	2.38	0.85/A
Cef+Sin A	175.16	74.11	29.89	13.68	0.17	0.18	5.86	0.36/S
Ami+Cur	45.47	566.21	8.74	83.26	0.19	0.15	5.20	0.34/S
Ami+Pip	45.47	>2048	15.68	350.32	0.34	0.17	2.90	0.52/A
Ami+Plu	45.47	>1280	24.42	794.95	0.54	0.62	1.86	1.16/I
Ami+Thy	45.47	464.84	15.58	162.53	0.34	0.35	2.92	0.69/A
Ami+Que	45.47	52.21	4.42	14.11	0.10	0.27	10.29	0.37/S
Ami+Sin A	45.47	74.11	1.38	5.37	0.03	0.07	32.95	0.10/S
Nor+Cur	106.95	566.21	7.77	42.95	0.07	0.08	13.76	0.15/S
Nor+Pip	106.95	>2048	49.68	485.05	0.46	0.24	2.15	0.70/A
Nor+Plu	106.95	>1280	50.53	848.84	0.47	0.66	2.12	1.14/I
Nor+Thy	106.95	464.84	30.32	104.42	0.28	0.22	3.53	0.51/A
Nor+Que	106.95	52.21	14.95	14.11	0.14	0.27	7.15	0.41/S
Nor+Sin A	106.95	74.11	8.42	13.89	0.08	0.19	12.70	0.27/S

ATB: antibiotics; NPs: natural products; MBIC: minimum biofilm inhibitory concentration; Cef: ceftazidime; Ami: amikacin; Nor: norfloxacin; Cur: curcumin; Pip: piperine; Plu: plumbagin; Thy: thymol; Que: quercetin; Sin A: sinapic acid; S: synergy; A: additivity; I: indifference.

**Table 4:** Mean of the minimum biofilm eradication concentration (MBEC), fractional inhibitory concentration (FIC), and fractional inhibitory concentration index (FICI) of antibiotics (ATB) and natural products (NPs) in combination against nineteen *P. aeruginosa* isolates.

Antimicrobial agent combination	MBEC (*μ*g/mL)				FIC		MBEC reduction fold of ATB	FICI/interpretation
	Alone		Combined					
	ATB	NPs	ATB	NPs	ATB	NPs		
Cef+Cur	815.16	>2048	390.74	687.16	0.48	0.34	2.09	0.81/A
Cef+Pip	815.16	>2048	481.68	917.68	0.59	0.45	1.69	1.04/I
Cef+Plu	815.16	>2048	323.37	1671.16	0.40	0.82	2.52	1.21/I
Cef+Thy	815.16	>2048	279.58	970.11	0.34	0.47	2.92	0.82/A
Cef+Que	815.16	195.37	245.89	97.68	0.30	0.50	3.32	0.80/A
Cef+Sin A	815.16	303.16	129.68	80.84	0.16	0.27	6.29	0.43/S
Ami+Cur	330.11	>2048	89.26	410.95	0.27	0.20	3.70	0.47/S
Ami+Pip	330.11	>2048	80.84	1293.47	0.24	0.63	4.08	0.88/A
Ami+Plu	330.11	>2048	119.58	1455.16	0.36	0.71	2.76	1.07/I
Ami+Thy	330.11	>2048	85.89	1212.63	0.26	0.59	3.84	0.85/A
Ami+Que	330.11	195.37	82.53	79.16	0.25	0.41	4.00	0.66/A
Ami+Sin A	330.11	303.16	10.39	35.95	0.03	0.12	31.77	0.15/S
Nor+Cur	619.79	>2048	35.16	250.05	0.06	0.12	17.63	0.18/S
Nor+Pip	619.79	>2048	195.37	1455.16	0.32	0.71	3.17	1.03/I
Nor+Plu	619.79	>2048	353.68	1455.16	0.57	0.71	1.75	1.28/I
Nor+Thy	619.79	>2048	202.11	632.32	0.33	0.31	3.07	0.63/A
Nor+Que	619.79	195.37	118.74	72.42	0.19	0.37	5.22	0.56/A
Nor+Sin A	619.79	303.16	60.63	70.74	0.10	0.23	10.22	0.33/S

ATB: antibiotics; NPs: natural products; MBEC: minimum biofilm eradication concentration; Cef: ceftazidime; Ami: amikacin; Nor: norfloxacin; Cur: curcumin; Pip: piperine; Plu: plumbagin; Thy: thymol; Que: quercetin; Sin A: sinapic acid; S: synergy; A: additivity; I: indifference.

## Data Availability

The data used to support the findings of this work are available from the corresponding author upon request.

## References

[1] Liu N., Pang X., Zhang H., Ji P. (2022). The cGAS-STING pathway in bacterial infection and bacterial immunity. *Frontiers in Immunology*.

[2] Yin R., Cheng J., Wang J., Li P., Lin J. (2022). Treatment of Pseudomonas aeruginosa infectious biofilms: challenges and strategies. *Frontiers in Microbiology*.

[3] Jurado-Martín I., Sainz-Mejías M., McClean S. (2021). Pseudomonas aeruginosa: an audacious pathogen with an adaptable arsenal of virulence factors. *International Journal of Molecular Sciences*.

[4] Pang Z., Raudonis R., Glick B. R., Lin T. J., Cheng Z. (2019). Antibiotic resistance in Pseudomonas aeruginosa: mechanisms and alternative therapeutic strategies. *Biotechnology Advances*.

[5] Beceiro A., Tomás M., Bou G. (2013). Antimicrobial resistance and virulence: a successful or deleterious association in the bacterial world?. *Clinical Microbiology Reviews*.

[6] Riquelme S. A., Liimatta K., Lung T. W. F. (2020). Pseudomonas aeruginosa utilizes host-derived itaconate to redirect its metabolism to promote biofilm formation. *Cell Metabolism*.

[7] Hall S., McDermott C., Anoopkumar-Dukie S. (2016). Cellular effects of pyocyanin, a secreted virulence factor of Pseudomonas aeruginosa. *Toxins*.

[8] Newman J. W., Floyd R. V., Fothergill J. L. (2017). The contribution of Pseudomonas aeruginosa virulence factors and host factors in the establishment of urinary tract infections. *FEMS Microbiology Letters*.

[9] Ndezo B. B., Lonkeng A. M., Tsopmene J. U., Dzoyem J. P. (2023). Biofilm Formation, Phospholipase and Proteinase Production in Cryptococcus neoformans Clinical Isolates and Susceptibility towards Some Bioactive Natural Products. *The Scientific World Journal*.

[10] Kuaté Tokam C. R., Bisso Ndezo B., Boulens N., Allémann E., Delie F., Dzoyem J. P. (2023). Antibiofilm activity and synergistic effects of thymol-loaded poly (lactic-co-glycolic acid) nanoparticles with amikacin against four Salmonella enterica serovars. *Canadian Journal of Infectious Diseases and Medical Microbiology*.

[11] Al-Wrafy F., Brzozowska E., Górska S., Gamian A. (2017). Pathogenic factors of Pseudomonas aeruginosa - the role of biofilm in pathogenicity and as a target for phage therapy. *Postepy Higieny i Medycyny Doswiadczalnej*.

[12] Schurek K. N., Breidenstein E. B. M., Hancock R. E. W. (2012). Pseudomonas aeruginosa: a persistent pathogen in cystic fibrosis and hospital-associated infections. *Antibiotic Discovery and Development*.

[13] Atanasov A. G., the International Natural Product Sciences Taskforce, Zotchev S. B., Dirsch V. M., Supuran C. T. (2021). Natural products in drug discovery: advances and opportunities. *Nature Reviews Drug Discovery*.

[14] Qureshi K. A., Imtiaz M., Parvez A. (2022). In vitro and in silico approaches for the evaluation of antimicrobial activity, time-kill kinetics, and anti-biofilm potential of thymoquinone (2-methyl-5-propan-2-ylcyclohexa-2, 5-diene-1,4-dione) against selected human pathogens. *Antibiotics*.

[15] Sun X., Zhang Y., Zhou Y. (2022). NPCDR: natural product-based drug combination and its disease-specific molecular regulation. *Nucleic Acids Research*.

[16] Hemaiswarya S., Kruthiventi A. K., Doble M. (2008). Synergism between natural products and antibiotics against infectious diseases. *Phytomedicine*.

[17] Tokam Kuaté C. R., Bisso Ndezo B., Dzoyem J. P. (2021). Synergistic antibiofilm effect of thymol and piperine in combination with aminoglycosides antibiotics against four Salmonella enterica serovars. *Evidence-Based Complementary and Alternative Medicine*.

[18] Bisso Ndezo B., Tokam Kuaté C. R., Dzoyem J. P. (2021). Synergistic antibiofilm efficacy of thymol and piperine in combination with three aminoglycoside antibiotics against Klebsiella pneumoniae biofilms. *Canadian Journal of Infectious Diseases and Medical Microbiology*.

[19] Guzzo F., Scognamiglio M., Fiorentino A., Buommino E., D’abrosca B. (2020). Plant derived natural products against Pseudomonas aeruginosa and Staphylococcus aureus: antibiofilm activity and molecular mechanisms. *Molecules*.

[20] Dzoyem J. P., McGaw L. J., Eloff J. N. (2014). In vitro antibacterial, antioxidant and cytotoxic activity of acetone leaf extracts of nine under-investigated Fabaceae tree species leads to potentially useful extracts in animal health and productivity. *BMC Complementary and Alternative Medicine*.

[21] Ndezo Bisso B., Tokam Kuaté C. R., Boulens N., Allémann E., Delie F., Dzoyem J. P. (2022). Antibiofilm synergistic activity of streptomycin in combination with thymol-loaded poly (lactic-co-glycolic acid) nanoparticles against Klebsiella pneumoniae isolates. *Evidence-Based Complementary and Alternative Medicine*.

[22] Klrmusaoaylu S., Kaşlkçl H. (2020). Identification of ica-dependent biofilm production by *Staphylococcus aureus* clinical isolates and antibiofilm effects of ascorbic acid against biofilm production. *Journal of Clinical Pathology*.

[23] Bellio P., Fagnani L., Nazzicone L., Celenza G. (2021). New and simplified method for drug combination studies by checkerboard assay. *MethodsX*.

[24] Alayande A. B., Aung M. M., Kim I. S. (2018). Correlation between quorum sensing signal molecules and Pseudomonas aeruginosa’s biofilm development and virulency. *Current Microbiology*.

[25] Abu El-Wafa W. M., Ahmed R. H., Ramadan M. A. H. (2020). Synergistic effects of pomegranate and rosemary extracts in combination with antibiotics against antibiotic resistance and biofilm formation of Pseudomonas aeruginosa. *Brazilian Journal of Microbiology*.

[26] Miklasińska-Majdanik M., Kępa M., Wojtyczka R. D., Idzik D., Wąsik T. J. (2018). Phenolic compounds diminish antibiotic resistance of Staphylococcus aureus clinical strains. *International Journal of Environmental Research and Public Health*.

[27] Ahmadu T., Ahmad K. (2021). An introduction to bioactive natural products and general applications. *Advanced Structured Materials*.

[28] Aka S. T., Haji S. H. (2015). Sub-MIC of antibiotics induced biofilm formation of Pseudomonas aeruginosa in the presence of chlorhexidine. *Brazilian Journal of Microbiology*.

[29] Thees A. V., Pietrosimone K. M., Melchiorre C. K. (2021). PmtA regulates pyocyanin expression and biofilm formation in Pseudomonas aeruginosa. *Frontiers in Microbiology*.

[30] Uruén C., Chopo-Escuin G., Tommassen J., Mainar-Jaime R. C., Arenas J. (2020). Biofilms as promoters of bacterial antibiotic resistance and tolerance. *Antibiotics*.

[31] Ciofu O., Rojo-Molinero E., Macià M. D., Oliver A. (2017). Antibiotic treatment of biofilm infections. *APMIS*.

[32] Pandi A., Kalappan V. M. (2021). Pharmacological and therapeutic applications of sinapic acid—an updated review. *Molecular Biology Reports*.

[33] Sanhueza L., Melo R., Montero R., Maisey K., Mendoza L., Wilkens M. (2017). Synergistic interactions between phenolic compounds identified in grape pomace extract with antibiotics of different classes against Staphylococcus aureus and Escherichia coli. *PLoS ONE*.

[34] Ayaz M., Ullah F., Sadiq A. (2019). Synergistic interactions of phytochemicals with antimicrobial agents: potential strategy to counteract drug resistance. *Chemico-Biological Interactions*.

[35] Álvarez-Martínez F. J., Barrajón-Catalán E., Micol V. (2020). Tackling antibiotic resistance with compounds of natural origin: a comprehensive review. *Biomedicines*.

[36] Liao C., Huang X., Wang Q., Yao D., Lu W. (2022). Virulence factors of Pseudomonas aeruginosa and antivirulence strategies to combat its drug resistance. *Frontiers in cellular and infection microbiology*.

